# Impact of Probiotic Administration on Serum C-Reactive Protein Concentrations: Systematic Review and Meta-Analysis of Randomized Control Trials

**DOI:** 10.3390/nu9010020

**Published:** 2017-01-03

**Authors:** Mohsen Mazidi, Peyman Rezaie, Gordon A. Ferns, Hassan Vatanparast

**Affiliations:** 1 Key State Laboratory of Molecular Developmental Biology, Institute of Genetics and Developmental Biology, Chinese Academy of Sciences, Beijing 100101, China; moshen@genetics.ac.cn; 2 Institute of Genetics and Developmental Biology, International College, University of Chinese Academy of Science, Beijing 100101, China; 3 Biochemistry and Nutrition Research Centre, School of Medicine, Mashhad University of Medical Science, Mashhad 42536, Iran; peymanrezaie.nutrition@gmail.com; 4 Division of Medical Education, Brighton and Sussex Medical School, Rm 342, Mayfield House, University of Brighton, Brighton BN1 9PH, UK; s.brown5@brighton.ac.uk; 5 College of Pharmacy and Nutrition, University of Saskatchewan, Health Sciences E-Wing, 104 Clinic Place, Saskatoon, SK S7N 2Z4, Canada

**Keywords:** meta-analysis, probiotic, C-reactive protein

## Abstract

We conducted this systematic review and meta-analysis of prospective studies to determine the effect of probiotic administration on serum C-reactive protein (CRP) concentrations. We searched PubMed-Medline, Web of Science, the Cochrane, and Google Scholar databases (until May 2016) to identify prospective studies evaluating the impact of probiotic administration on CRP. We used a random effects models and generic inverse variance methods to synthesize quantitative data, followed by a leave-one-out method for sensitivity analysis. The systematic review registration number was: CRD42016039457. From a total of 425 entries identified via searches, 20 studies were included in the final analysis. The meta-analysis indicated a significant reduction in serum CRP following probiotic administration with a weighted mean difference (WMD) of −1.35 mg/L, (95% confidence interval (CI) −2.15 to −0.55, *I*^2^ 65.1%). The WMDs for interleukin 10 (IL10) was −1.65 pg/dL, (95% CI −3.45 to 0.14, *I*^2^ 3.1%), and −0.45 pg/mL, (95% CI −1.38 to 0.48, *I*^2^ 10.2%) for tumor necrosis factor alpha (TNF-α). These findings were robust in sensitivity analyses. This meta-analysis suggests that probiotic administration may significantly reduce serum CRP while having no significant effect on serum IL10 and TNF-α.

## 1. Introduction

Probiotics have been described as ‘live microorganisms which, when administered in adequate amounts, have a health benefit on the host’ [1,2] through their impact on the intestinal tract. Probiotics are now extensively consumed in the form of fermented milk products such as yogurt or as a freeze-dried culture [3,4,5]. The main probiotic bacteria related to dairy products include *Lactobacillus acidophilus*, *Lactobacillus casei*, and *Bifidobacteria* [2,4]. It is suggested that probiotics not only improve the balance of gut microbiota in favor of the healthy bacteria but are also helpful in either preventing or improving the outcomes of a number of health conditions such as obesity, insulin resistance, type 2 diabetes, and non-alcoholic fatty liver disease [6,7]. Some beneficial effects of probiotics include modulation of intestinal microbiota, strengthening of the epithelial barrier, and immunomodulation [8].

Serum C-reactive protein (CRP) is a marker of systemic inflammation, and is elevated in the presence of chronic conditions, including cardiovascular diseases (CVD) [9,10], obesity [11], type 2 diabetes [12], and several components of the metabolic syndrome [10,13], including high blood pressure [14], high waist circumference [15], fasting blood glucose [16,17,18], low serum high-density lipoprotein cholesterol, and raised triacylglycerol [19]. The anti-inflammatory properties of some strains of probiotics are thought to act by reducing mucosal inflammation via modulation of cytokine levels, and other inflammatory mediators are reported in some in vitro and in vivo studies [6,20]. Studies suggest the consumption of probiotic yogurt containing *L. acidophilus* and *Bifidobacterium animalis* in pregnant women for nine weeks led to a reduction in serum high sensitivity (hs)-CRP as the same probiotic supplementation in colorectal cancer, autoimmune, and chronic kidney disease [21,22,23]. The decreased inflammation and oxidative stress due to probiotics might be due to their effects on increasing glutathione (GSH) levels and scavenging superoxide and hydroxyl radicals, decreasing the expression of interleukin-6 (IL-6) in adipocytes, and decreasing adiposity [24,25]. However, some studies have reported a non-significant effect of probiotic supplementation on serum hs-CRP level [22,26]. Moreover, there are limitations of the existing studies such as small sample size, issues with research design, and subject traits (gender, ethnicity, age, etc.). These problems limit the ability to draw reliable conclusions. On the other hand, dietary supplementation with probiotics can have different effects on some indices of inflammatory and anti-inflammatory indexes. Meta-analysis may overcome these limitations by increasing the effective sample size. Hence, the present study aimed to resolve the uncertainty about the impact of probiotics on serum hs-CRP as an indicator of inflammation by systematically reviewing the literature and performing meta-analysis of all randomized control trials investigating the effects of probiotic supplementation on serum hs-CRP levels as one of the measures of inflammation related to disease outcome.

## 2. Materials and Methods

### 2.1. Literature Search Strategy

We designed the present study according to the Preferred Reporting Items for Systematic Reviews and Meta-Analyses (PRISMA) Guidelines [27,28,29]. We registered the study protocol with the International Prospective Register of Systematic Reviews, PROSPERO (registration No. CRD42016039457). The primary exposure of interest was probiotic administration while the primary outcome of interest was changes in CRP levels subsequent to probiotic administration. We also evaluated the impact of probiotics on interleukin 10 (IL10), tumor necrosis factor alpha (TNF-α), interleukin 1β (IL1β), serum triglycerides (TG), serum total cholesterol (TC), serum high density lipoproteins (HDL), serum low density lipoproteins (LDL), and fasting blood glucose (FBG). We searched multiple databases, including PUBMED/Medline, Cochrane Central Register of Controlled Trials (CCTR), Cochrane Database of Systematic Reviews (CDSR), MEDLINE and Web of Science, until May 2016 using a combination of search terms available in the Supplementary Materials Table S1. The wild-card term ‘*’ was used to increase the sensitivity of the search strategy. No language restriction was applied. We hand searched the reference list of qualified articles and conducted email correspondence with authors for additional data where relevant.

### 2.2. Selection Criteria

**Figure 1:**
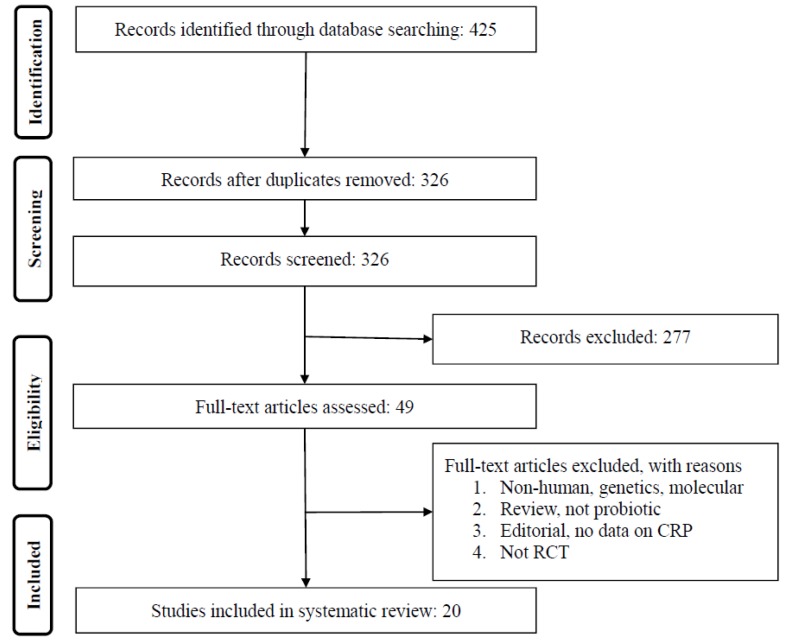
Preferred Reporting Items for Systematic Reviews and Meta-Analyses (PRISMA) flow chart for the selection of studies. CRP, C-reactive protein; RCT, randomized control trial.

We included all randomized control trials (RCTs) studies evaluating the effect of probiotic supplementation on the outcome of interest. The inclusion criteria were as follows: (1) a controlled trial with either parallel or crossover design; (2) presentation of satisfactory information on primary outcome at baseline and at the end of follow-up in each group or the net change values; (3) prospective studies of patients treated with probiotic supplementation compared to a control group (either no probiotic supplementation or placebo). Exclusion criteria were as follows: (i) non-clinical studies; (ii) observational studies with case–control, cross-sectional or cohort design; and (iii) studies that did not provide mean (or median) plasma concentrations of the outcomes of interest at baseline and/or the end of the trial. We also excluded narrative reviews, papers in a language other than English, comments, opinion pieces, methodological papers, editorials, letters, or any other publications lacking primary data and/or explicit method descriptions. Duplicate studies were removed by a screening of titles and abstracts by two reviewers. To prevent potential bias, the reviewers were blinded to the names, qualifications, and institutional affiliations of the study authors. The agreement between the reviewers was excellent (Kappa index: 0.88; *p* < 0.001). Disagreements were resolved at a meeting between reviewers prior to the selected articles being retrieved (Figure 1).

### 2.3. Data Extraction and Management

The full text of studies meeting the inclusion criteria was retrieved and screened to determine eligibility by two reviewers (Mohsen Mazidi, Peyman Rezaie). Following assessment of methodological quality, the two reviewers extracted data using a purpose-designed data extraction form and independently summarized what they considered to be the most outstanding results from each study. These summaries were compared and any differences of opinion resolved by discussion and consultation with a third reviewer. Any further necessary calculations on study data were conducted by the first reviewer and checked by the second reviewer. Descriptive data extracted included the first author’s name, reference, country, study design, probiotics, delivery method, duration (weeks), age (years), type of CRP assay used, background disease and sample size.

### 2.4. Quality Assessment

We used the Cochrane criteria to systematically assess bias in the eligible RCTs [30]. The items used for the assessment of each study were (i) adequacy of random sequence generation; (ii) allocation concealment; (iii) blinding of participants; (iv) personnel and outcome assessment; (v) handling of drop-outs (incomplete outcome data); (vi) selective outcome reporting; and (vii) other potential sources of bias. A judgment of ‘yes’ indicated low risk of bias, while ‘no’ indicated a high risk of bias, taking into account the recommendations of the Cochrane Handbook. We labeled uncertain or unknown risk of bias as “unclear”.

### 2.5. Data Synthesis

Following the recommendation of the Cochrane Handbook, to calculate the effect size, we used the mean change from baseline to the end point in the concentrations and standard deviation (SD) of the variables of interest for both control and intervention groups [30,31]. In brief, we calculated the net changes in measurements (change scores) as the measure at the end of follow-up − measure at baseline. For RCTs, change scores were calculated as (measure at the end of follow-up in the treatment group − measure at baseline in the treatment group) − (measure at the end of follow-up in the control group − measure at baseline in the control group). We used the following formula to calculate standard deviation (SD) in situations where only the standard error of the mean (SEM) was available: SD = SEM × square root (*n*), where n is the number of subjects [17]. If the outcome measures were reported in median and range (or 95% confidence interval (CI)), we estimated the mean and standard SD values using the method described by Hozo et al. [32]. Blood lipid and glucose levels were collated in mmol/L; a multiplication factor of 0.0259, 0.0113, or 0.0555 was used to convert cholesterol (total cholesterol, HDL-C or LDL-C), triglycerides and glucose levels respectively from mg/dL to mmol/L as appropriate [17].

We used a random effects model (using the DerSimonian–Laird method) and the generic inverse variance method to compensate for the heterogeneity of studies regarding demographic characteristics of populations being studied [33,34]. Heterogeneity was quantitatively assessed using an *I*^2^ index. *I*^2^ values <50% and ≥50% corresponded with the use of a fixed-effects or random-effects model, respectively. Effect sizes are expressed as the weighted mean difference (WMD) and 95% confidence interval (CI). To determine the influence of each study on the overall effect size, a sensitivity analysis was conducted using the leave-one-out method (i.e., removing one study each time and repeating the analysis). The sensitivity analysis involved repeating the meta-analysis and substituting alternative decisions or ranges of values for decisions that were arbitrary or unclear [35,36,37].

### 2.6. Publication Bias

We visually inspected the Begg’s funnel plot asymmetry, Begg’s rank correlation, and Egger’s weighted regression tests to evaluate the potential publication bias [18]. This step was followed by adjusting the analysis for the effects of publication bias using the Duval & Tweedie ‘trim and fill’ and ‘fail-safe N’ methods [38]. We conducted the meta-analysis using Comprehensive Meta-Analysis (CMA) V3 software (Biostat, Englewood, NJ, USA) [17,39].

## 3. Results

### 3.1. Summary of Searches and Study Selection Process

We identified a total of 425 citations, of which 326 records remained after removing duplicates. After screening via titles and abstracts, 49 articles remained for further evaluation, of which 29 were excluded for the following reasons: non-human studies, genetic, or molecular studies (*n* = 15); reviews or editorial articles (*n* = 5); or short follow-up duration (*n* = 9); see Figure 1. Finally, 20 studies met all inclusion criteria for the meta-analysis. All these studies were in English. We found five studies in non-English languages. These studies were excluded at the first step of the study selection procedure.

### 3.2. Risk of Bias Assessment

There was a lack of information about blinding of outcome assessment (*n* = 2) and blinding of participants and personnel (*n* = 2); however, all evaluated studies had a low risk of bias according to selective outcome reporting. Details of the quality of bias assessment are shown in Supplementary Materials Table S2.

### 3.3. Characteristics of the Eligible Studies

The characteristics of the included studies are summarized in Table 1. These studies were published between 2003 and 2015 from fifteen countries including Iran (four studies), India (three studies), Finland (two studies), and one study from each of the following countries: the United States of America, Denmark, New Zealand, Austria, Sweden, Canada, Turkey, Norway, China, Spain, and one multi-country study. Participants in two studies were only females [6,40], while the proportion of men in other studies ranged from 9% [40] to 95% [41]. The mean age of participants fluctuated from 6 months [42] to 85 years [43]. The duration of follow-up across studies ranged from 7 days [42,44] to 6 months [45]. A nasogastric tube was used as a method of delivery of probiotic in two studies [44,46] while the other studies used oral supplementation as the method of delivery.

### 3.4. Pooled Estimate of the Effect of Probiotic Administration on CRP

**Figure 2:**
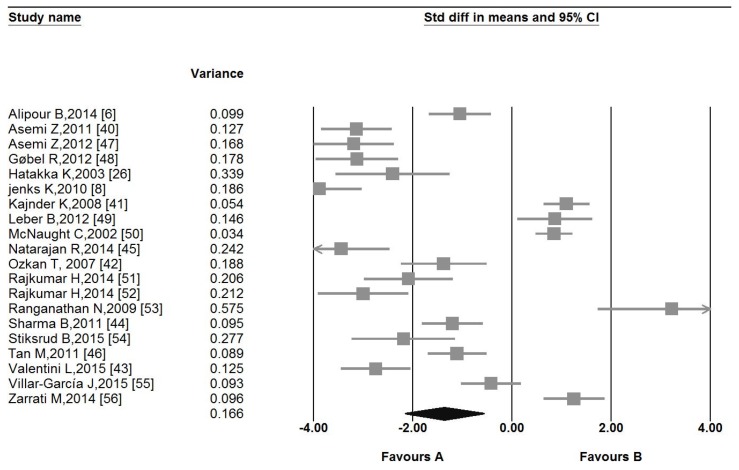
Forest plot displaying weighted mean difference and 95% confidence intervals for the impact of probiotic administration on C-reactive protein (CRP) levels.

The pooled estimate (weighted mean difference) of the effect of probiotic administration on CRP levels was −1.35 mg/L, (95% CI −2.15 to −0.55, *I*^2^ 65.1%) across all studies (Figure 2). 

Results on the effect of probiotic administration on the other inflammatory, anti-inflammatory, lipid profile, and glycemia measurements are presented in Table 2.

### 3.5. Sensitivity Analysis

In leave-one-out sensitivity analyses, the pooled effect estimates remained similar across all studies which confirm that the significant difference between the studied groups is the overall effect of all included studies. Analysis showed that if we only pool the studies with the enzyme-linked immunosorbent assay (ELISA) method (CRP measurement), the heterogeneity is reduced to 52.1%.

### 3.6. Publication Bias

**Figure 3:**
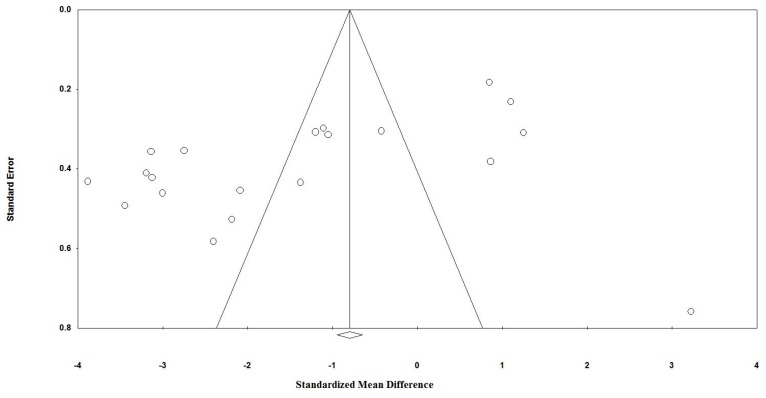
Funnel plots detailing publication bias in the studies selected for analysis. Open circles represent observed published studies; open diamond represents observed effect size.

**Figure 4:**
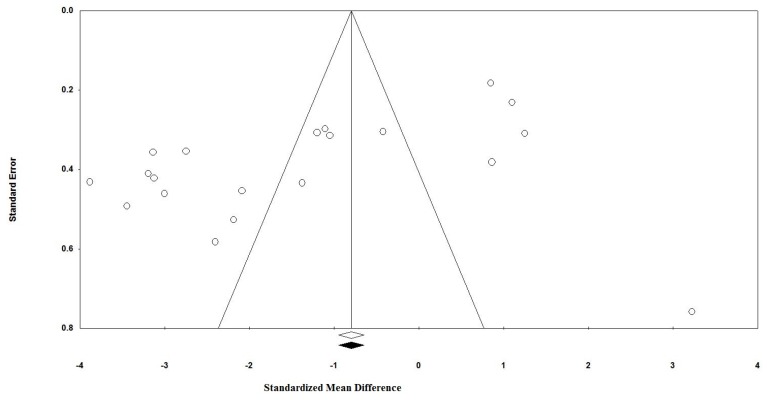
Trim and fill method was used to impute for potentially missing studies, no potentially missing study was imputed in funnel plot. Open circles represent observed published studies; open diamond represents observed effect size; closed diamond represents imputed effect size.

Visual inspection of funnel plot asymmetry indicated a potential publication bias for the comparison of plasma CRP levels between probiotic administered groups and placebo groups (Figure 3). Further, the presence of publication bias was suggested by Egger’s linear regression (intercept = −9.39, standard error = 3.04; 95% CI = −15.80, 2.99, *t* = 3.08, degree of freedom (*df*) = 18.00, two-tailed *p* < 0.001) but Begg’s rank correlation test was not indicative of a publication bias (Kendall’s Tau with continuity correction = −0.28, *z* = 1.75, two-tailed *p* = 0.079). After adjustment of effect size for potential publication bias using the ‘trim and fill’ correction, no potentially missing study was imputed in the funnel plot (WMD 1.35 mg/dL, 95% CI −2.15 to −0.55) (Figure 4). The ‘fail-safe N’ test showed that 1003 studies would be needed to bring the WMD down to a non-significant (*p* > 0.05) value.

## 4. Discussion

This meta-analysis suggests that probiotic administration may reduce serum CRP levels. A strong relationship has been reported between the level of oxidative stress and inflammatory markers and risk of cardiovascular disease [57,58]. Along with other pathophysiological complications of oxidative stress, inflammation is associated with insulin resistance. This, in turn, causes diminished glucose uptake and disposal in peripheral tissues and increased glucose production in the liver [57,58].

Similar to our findings, Asemi et al. demonstrated that consumption of probiotic supplements results in a significant reduction in serum hs-CRP levels compared with placebo [24]. Moreover, consumption of a combination of *L. casei*, *B. breve*, and prebiotic galacto oligosaccharides [59] as well as *B. longum* [60] in immunocompromised patients has been found to decrease serum hs-CRP levels in only three of the studies that measured hs-CRP. The initial hs-CRP levels were well below the cutoff of ≥10 mg/L as an indicator of acute inflammation (Supplementary Materials Table S1). The anti-inflammatory effect of *Lactobacillus reuteri* and the anti-oxidant and anti-inflammatory effects of *L. plantarum* are among studies in which the impact of probiotics on the management of diabetes has been investigated [61]. In animal studies, Yadav et al. stated that the feeding of probiotic dahi (yogurt) containing 108 strains of *L. acidophilus* and *L. casei* delayed the onset of glucose intolerance, hyperglycemia, hyperinsulinemia, dyslipidemia, and oxidative stress in fructose-induced type 2 diabetes rats.

Several mechanisms are suggested to explain the effects of probiotics on serum hs-CRP levels including the effects of short-chain fatty acids that are produced from probiotics in the colon [24,25]. This consequently results in decreased enzymatic synthesis of hepatic hs-CRP. The decreased serum hs-CRP levels might also result from decreased expression of IL-6. It has also been suggested that decreased inflammation and oxidative stress by probiotics consumption might be due to their effects on increasing glutathione (GSH) levels, scavenging superoxide and hydroxyl radicals, decreasing expression of interleukin-6 (IL-6) in adipocytes, and decreasing adiposity [24,25]. In contrast, Hattakka et al. have reported that probiotic supplementation had no significant effect on the serum concentrations of several cytokines [6,26]. Furthermore, Shoaei et al. reported an 8-week multispecies probiotics supplementation had a non-significant beneficial effect on pancreatic β-cell function and hs-CRP in polycystic ovary syndrome (PCOS) patients [22]. The disagreement between the results of the previously conducted clinical trials may be in part due to the different strains of probiotics and dose of the probiotic administered. Some in vitro studies have indicated that certain effects are seen only when low doses of probiotics are applied, and that high doses may cause opposite effects compared to those obtained at lower doses [6,62]. Several human studies have also recommended that lower doses of probiotics may be more effective and improve cellular immunity to the preferred extent [63].

Several mechanisms have been suggested concerning the impact of probiotics on inflammation and inflammatory factors. Probiotics can prevent or repair the ‘leaky’ epithelial barriers and indirectly affect the inflammatory response by opposing the source of pro-inflammatory impetuses associated with low-grade endotoxemia [2,64]. Further, probiotics increase production of short-chain fatty acids (SCFA) such as butyrate which has an anti-inflammatory function. They also enhance synthesis of antimicrobial peptides that influence inflammation resolution pathways in the mucosa [64,65,66]. Probiotics can act as ligands for innate immune system receptors and directly affect pro-inflammatory pathways. They also stimulate the differentiation and activity of immune cells such as dendritic cells and T-cells and consequently enhance production of some regulatory cytokines [65,66].

As an important point, we also need to consider the effect size for each of the outcomes of interest and compare it with the normal range of the value to understand the magnitude of changes.

There are some potential limitations in our analysis that need to be addressed. First, as with any meta-analysis, internal validity relies on the quality of individual studies. Most of the included studies had relatively medium sample sizes, potentially leading to overestimation of treatment effects; smaller trials might be methodologically less robust and more prone to report larger effect sizes [67,68]. Another limitation is the variability in the type of CRP assays across the studies under this review. Only three of these have applied a high-sensitivity (hs) CRP assay (Supplementary Materails Table S1). Although the hs-CRP values in those studies were ≥10 mg/L as the cut-off for acute inflammation, our results should be interpreted with caution. The studies that met the inclusion criteria and were analyzed are also heterogeneous regarding the main health outcomes under the studies and the type of CRP assays. Only three of them applied a high-sensitivity CRP assay (Supplementary Materials Table S1). Hence, our results should be interpreted with caution.

## 5. Conclusions

Our meta-analysis suggests consuming probiotics appears to be beneficial by decreasing some inflammatory cytokines and biomarkers that may play a role in the development of cardiovascular disease and type 2 diabetes mellitus. More clinical trials with longer duration and larger sample sizes and consistency in terms of the laboratory measurements are still needed to explore the exact dose and strains of probiotic supplement in each specific disease. Although we found a protective effect of probiotics on CRP, these results should be interpreted with caution due to the (i) heterogeneity of health outcomes; (ii) variability in the assays under the studies included in this review; and (iii) complexity of the pathways in which gut microbiota play a role in the inflammation and anti-inflammation balance in different diseases.

## Figures and Tables

**Table 1:** General characteristics of the studies included in meta-analysis.

First Author, Reference	Country	Total Sample Size (% Female)	Study Design	Probiotics Features	Delivery Method	Duration (Weeks)	Age (Years)	C-reactive Protein (CRP) Assay	Background Disease	Sample Size
Alipour B, 2014 [6]	Iran	44 (100%)	Randomized, double-blind, placebo-controlled trial,	10^8^ colony forming units (CFU) of *L. casei 01* and maltodextrin	Orally	8 weeks	Test: 44.29; Control: 41.14	Turbidometric assay	Rheumatoid arthritis	30
Asemi Z, 2011 [40]	Iran	35 (100%)	Prospective, randomized, single-blinded clinical trial	Probiotic yogurt enriched with *Lactobacillus acidophilus* and *Bifidobacterium* animalis	Orally	9 weeks	Test: 24.2; Control: 25.7	Enzyme-linked immunosorbent assay (ELISA)	Healthy	37
Asemi Z, 2013 [47]	Iran	54 (70%)	Randomized double-blinded controlled clinical trial	*Lactobacillus acidophilus* (2 × 10^9^ CFU), *L. casei* (7 × 10^9^ CFU), *L. rhamnosus* (1.5 × 10^9^ CFU), *L. bulgaricus* (2 × 10^8^ CFU), *Bifidobacterium breve* (2 × 10^10^ CFU), *B. longum* (7 × 10^9^ CFU), *Streptococcus thermophilus* (1.5 × 10^9^ CFU)	Orally	8 weeks	Test: 52.59; Control: 50.51	ELISA	Diabetic patients	54
Gobel R, 2012 [48]	Denmark	50 (Test: 52%; Control: 59.2)	Double-blinded, randomized, placebo controlled intervention study	*L. salivarius* (10^10^ CFU)	Orally	12 weeks	12–15	Specific high-sensitivity CRP	Obese	50
Hattakka K, 2003 [26]	Finland	21 (Test: 100%; Control: 61.5%)	Randomized, double-blind, placebo-controlled study	*Lactobacillus rhamnosus,* 5 × 10^9^ CFU	Orally	12 weeks	Test: 50; Control: 53	-------	Rheumatoid arthritis	21
Jenks K, 2010 [8]	New Zealand	62 (Test: 41%; Control: 32%)	Randomized controlled trial	*Streptococcus salivarius, Bifidobacterium lactis,* and *Lactobacillus acidophilus*	Orally	12 weeks	Test: 45.5; Control: 41.1	ELISA	Spondyloarthritis	63
Kajander K, 2007 [41]	Finland	86 (Test: 5%; Control: 91%)	Randomized double-blind, placebo-controlled,	*L. rhamnosus GG, L. rhamnosus, P. freudenreichii* ssp. *shermanii JS*, 10^7^ CFU	Orally	5 months	Test: 50; Control: 46	Particle-enhanced immunoturbidimetric assay	Irritable bowel syndrome patients	86
Leber B, 2012 [49]	Austria	30 (Test: 30.7%; Control: 40%)	An open label, randomized pilot study	*L. casei Shirota*, 6.5 × 10^9^ CFU	Orally	3 months	Test: 51.5; Control: 54.5	-----	Metabolic syndrome	28
Mc Naught C, 2002 [50]	Sweden	130 (Test: 39%; Control: 44.6%)	Prospective and randomized	*Lactobacillus plantarum*, 5 × 10^7^ CFU	Orally	2 weeks	Test: 68; Control: 69	-----	Surgical patients	129
Natarajan R, 2014 [45]	USA	41 (16.7%)	Randomized, double-blind, placebo-controlled crossover study	*Renadyl*	Orally	6 months	29–79	-----	End-stage renal disease	-----
Ozkan T, 2007 [42]	Turkey	27 (44.4%)	Prospectively	*Saccharomyces boulardii*	Orally	7 days	6 months to 10 years	ELISA	Healthy	27
Rajkumar H, 2014 [51]	India	30 (53.8%)	A randomized controlled single-blind pilot study	*Lactobacillus salivarius,* 2 × 10^9^ CFU	Orally	6 weeks	20–25	dbc-hs Krishgen	Healthy	45
Rajkumar H, 2014 [52]	India	40 (50%)	A randomized, controlled trial	*Lyophilized Bifidobacteria, Lactobacilli, and Streptococcus thermophilus*, 112.5 × 10^9^ CFU	Orally	6 weeks	40–60	dbc-hs Krishgen	Healthy	60
Ranganathan N, 2009 [53]	Canada	16 (30.7%)	Pilot scale trial	*L. acidophilus B. longum and S. thermophilus*, 1.5 × 10^10^ CFU	Orally	6 months	54	-----	Chronic kidney disease	16
Sharma B, 2011 [44]	India	50 (Test: 57.6%; Control: 50%)	A double-blind randomized placebo-controlled trial	*Lactobacillus acidophilus*, *Bifidobacterium longus*, *Bifidobacterium bifidum*, and *Bifidobacterium infantalis*	Oral, nasojejunal, alternatively, nasogastric	7 days	Test: 40.19; Control: 41	ELISA	Acute pancreatitis	50
Stiksrud B, 2015 [54]	Norway	24 (Test: 28.6%; Control: 100%)	Randomized in a double-blind	*Lactobacillus rhamnosus, Bifidobacterium animalis* subsp. *lactis* and *Lactobacillus acidophilus La-5*, 10^8^ CFU	Orally	8 weeks	Test: 50.3; Control: 52.5	-----	Patients on antiretroviral therapy	32
Tan M, 2011 [46]	China	52 (Test: 26.9%; Control: 19.2%)	A prospective, randomized pilot study	*Bifidobacterium longum*, *Lactobacillus bulgaricus*, and *Streptococcus thermophilus*, 10^9^ CFU	nasogastric tube	21 days	Test: 40.5; Control: 40.8	ELISA	Traumatic brain injury	26
Valentini L, 2015 [43]	France, Germany, Italy	62 (53.2%)	Randomized controlled trial	*Bifidobacterium infantis, Bifidobacterium longum, Bifidobacterium breve, Lactobacillus acidophilus, Lactobacillus delbrückii* ssp. *bulgaricus, Lactobacillus paracasei, Lactobacillus plantarum*	Orally	8 weeks	65–85	ELISA	Healthy	62
Villar Garcia J, 2015 [55]	Spain	44 (Test: 9.1%; Control: 22.7%)	A single-center, randomized, double-blind, placebo-controlled pilot study	*S. boulardii*, 6 × 10^7^ CFU	Orally	12 weeks	Test: 49.45; Control: 45.5	Immulite chemiluminescent immunometric assay	HIV-1–infected patients with virologic suppression	44
Zarrati M, 2014 [56]	Iran	50 (68%)	Randomized double-blind controlled clinical trial	*Lactobacillus acidophilus*, *Lactobacillus casei*, *Bifidobacterium lactis*, 10^8^ CFU	Orally	8 weeks	20–50	-----	Overweight and obese individuals	75

**Table 2:** Effect of probiotic administration on the other inflammatory, anti-inflammatory, lipid profile and glycemia measurements.

Factors	Results of the Pooled Estimate
IL10	−1.65 pg/dL, (95% CI −3.45 to 0.14)
TNF-α	−0.45 pg/mL, (95% CI −1.38 to 0.48)
IL1β	−1.07 pg/dL, (95% CI −1.55 to −0.59)
TG	−0.92 mg/dL, (95% CI −1.22 to −0.62)
TC	−0.58 mg/dL, (95% CI −0.84 to −0.32)
LDL	−1.36 mg/dL, (95% CI −1.70 to −1.02)
HDL	0.51 mg/dL, (95% CI 0.19 to 0.83)
FBG	−0.75 mg/dL, (95% CI −1.11 to −0.38)

IL10, interlukin 10; TNF-α, tumor necrosis factor alpha; IL1β, interleukin 1β; TG, triglycerides; TC, total cholesterol; LDL, low density lipoproteins; HDL, high density lipoproteins; FBG, fasting blood glucose.

## Supplementary Materials

The following are available online at http://www.mdpi.com/2072-6643/9/1/20/s1, Table S1: Full Search terms and strategy used for systematically reviewing the articles, Table S2: Quality of bias assessment of the included studies according to the Cochrane guidelines.
