# Supplementary material for: Impact of Probiotic Administration on Serum C-Reactive Protein Concentrations: Systematic Review and Meta-Analysis of Randomized Control Trials
Source: Nutrients. 2017 Jan 3;9(1):20. doi: 10.3390/nu9010020 (PMC5295064; doi:10.3390/nu9010020)
Supplement: Supplementary file 1 [file nutrients-09-00020-s001.docx]

**Supplementary Materials: Impact of Probiotic Administration on Serum C-Reactive Protein Concentrations: Systematic Review and
Meta-Analysis of Randomized Control Trials**

Mohsen Mazidi, Peyman Rezaie, Gordon A. Ferns and Hassan Vatanparast

**Table S1.** Full search terms and strategy used for systematically reviewing the articles.

| **No.** | **Concept** | **Search Terms** |
| --- | --- | --- |
| **#1** | **Probiotic** | probiotic (Title/Abstract) OR streptococcus (Title/Abstract)  OR lactobacill (Title/Abstract) OR saccharomyces (Title/Abstract)  OR enterococcus (Title/Abstract) OR lactococcus (Title/Abstract)  OR bifidobacter (Title/Abstract) OR VSL#3 (Title/Abstract)  OR yogurt (Title/Abstract) OR yoghurt (Title/Abstract)  OR ‘‘fermented milk’’ (Title/Abstract) OR ‘‘sour milk’’ (Title/Abstract) |
| **#2** | **C-reactive protein** | “high sensitivity C-reactive protein” (MeSH Terms)  OR “high-sensitivity C-reactive protein” (MeSH Terms)  OR “C-reactive protein” (MeSH Terms)  OR “high-sensitive C-reactive protein” (MeSH Terms)  OR “high sensitive C-reactive protein” (MeSH Terms)  OR “CRP (Title/Abstract) OR “hsCRP” (Title/Abstract) |
| **3** | **Combination** | 1 AND 2 |

**Table S2.** Quality of bias assessment of the included studies according to the Cochrane guidelines.

| **First Author, Year of Publication** | **Random Sequence Generation** | **Allocation Concealment** | **Selective Reporting** | **Blinding of Participants and Personnel** | **Blinding of Outcome Assessment** | **Incomplete Outcome Data** | **Other Bias** |
| --- | --- | --- | --- | --- | --- | --- | --- |
| Alipour B, 2014 [1] | L | L | L | H | L | H | L |
| Asemi Z, 2011 [2] | L | L | L | L | L | L | L |
| Asemi Z, 2013 [3] | L | H | L | L | L | L | L |
| Gobel R, 2012 [4] | L | L | L | L | L | L | H |
| Hattakka K, 2003 [5] | L | L | L | L | L | H | L |
| Jenks K, 2010 [6] | L | L | L | L | L | L | L |
| Kajander K, 2007 [7] | L | L | L | L | L | H | L |
| Leber B, 2012 [8] | L | H | L | L | U | L | L |
| Mc Naught C, 2002 [9] | L | L | L | L | L | L | L |
| Natarajan R, 2014 [10] | L | L | H | L | L | L | L |
| Ozkan T, 2007 [11] | L | L | L | L | L | L | L |
| Rajkumar H, 2014 [12] | L | L | L | U | L | L | L |
| Rajkumar H, 2014 [13] | L | L | L | L | L | L | L |
| Ranganathan N, 2009 [14] | L | L | H | L | L | L | L |
| Sharma B, 2011 [15] | L | L | L | L | L | H | L |
| Stiksrud B, 2015 [16] | L | L | L | L | L | L | L |
| Tan M, 2011 [17] | L | L | L | L | L | H | L |
| Valentini L, 2015 [18] | L | L | L | L | L | L | L |
| Villar Garcia J, 2015 [19] | L | L | L | L | L | L | L |
| Zarrati M, 2014 [20] | L | L | H | L | L | L | L |

L, low risk of bias; H, high risk of bias; U, unclear risk of bias.

References

1. Alipour, B.; Homayouni-Rad, A.; Vaghef-Mehrabany, E.; Sharif, S.K.; Vaghef-Mehrabany, L.; Asghari-Jafarabadi, M.; Nakhjavani, M.R.; Mohtadi-Nia, J. Effects of *Lactobacillus* casei supplementation on disease activity and inflammatory cytokines in rheumatoid arthritis patients: A randomized double-blind clinical trial. *Int. J. Rheum. Dis.* **2014**, *17*, 519–527.
2. Asemi, Z.; Jazayeri, S.; Najafi, M.; Samimi, M.; Mofid, V.; Shidfar, F.; Foroushani, A.R.; Shahaboddin, M.E. Effects of daily consumption of probiotic yoghurt on inflammatory factors in pregnant women: A randomized controlled trial. *Pak. J. Biol. Sci.* **2011**, *14*, 476–482.
3. Asemi, Z.; Hashemi, T.; Karamali, M.; Samimi, M.; Esmaillzadeh, A. Effects of vitamin D supplementation on glucose metabolism, lipid concentrations, inflammation, and oxidative stress in gestational diabetes: A double-blind randomized controlled clinical trial. *Am. J. Clin. Nutr.* **2013**, *98*, 1425–1432.
4. Gobel, R.J.; Larsen, N.; Jakobsen, M.; Molgaard, C.; Michaelsen, K.F. Probiotics to adolescents with obesity: Effects on inflammation and metabolic syndrome. *J. Pediatr. Gastroenterol. Nutr.* **2012**, *55*, 673–678.
5. Hatakka, K.; Martio, J.; Korpela, M.; Herranen, M.; Poussa, T.; Laasanen, T.; Saxelin, M.; Vapaatalo, H.; Moilanen, E.; Korpela, R. Effects of probiotic therapy on the activity and activation of mild rheumatoid arthritis—A pilot study. *Scand. J. Rheumatol.* **2003**, *32*, 211–215.
6. Jenks, K.; Stebbings, S.; Burton, J.; Schultz, M.; Herbison, P.; Highton, J. Probiotic therapy for the treatment of spondyloarthritis: A randomized controlled trial. *J. Rheumatol.* **2010**, *37*, 2118–2125.
7. Kajander, K.; Myllyluoma, E.; Rajilic-Stojanovic, M.; Kyronpalo, S.; Rasmussen, M.; Jarvenpaa, S.; Zoetendal, E.G.; de Vos, W.M.; Vapaatalo, H.; Korpela, R. Clinical trial: Multispecies probiotic supplementation alleviates the symptoms of irritable bowel syndrome and stabilizes intestinal microbiota. *Aliment. Pharmacol. Ther.* **2008**, *27*, 48–57.
8. Leber, B.; Tripolt, N.J.; Blattl, D.; Eder, M.; Wascher, T.C.; Pieber, T.R.; Stauber, R.; Sourij, H.; Oettl, K.; Stadlbauer, V. The influence of probiotic supplementation on gut permeability in patients with metabolic syndrome: An open label, randomized pilot study. *Eur. J. Clin. Nutr.* **2012**, *66*, 1110–1115.
9. McNaught, C.E.; Woodcock, N.P.; MacFie, J.; Mitchell, C.J. A prospective randomised study of the probiotic *Lactobacillus* plantarum 299v on indices of gut barrier function in elective surgical patients. *Gut* **2002**, *51*, 827–831.
10. Natarajan, R.; Pechenyak, B. Randomized controlled trial of strain-specific probiotic formulation (renadyl) in dialysis patients. *Biomed. Res. Int.* **2014**, *2014*, 568571.
11. Ozkan, T.B.; Sahin, E.; Erdemir, G.; Budak, F. Effect of saccharomyces boulardii in children with acute gastroenteritis and its relationship to the immune response. *J. Int. Med. Res.* **2007**, *35*, 201–212.
12. Rajkumar, H.; Kumar, M.; Das, N.; Kumar, S.N.; Challa, H.R.; Nagpal, R. Effect of probiotic *Lactobacillus* *Salivarius* ubl s22 and prebiotic fructo-oligosaccharide on serum lipids, inflammatory markers, insulin sensitivity, and gut bacteria in healthy young volunteers: A randomized controlled single-blind pilot study. *J. Cardiovasc. Pharmacol. Ther.* **2015**, *20*, 289–298.
13. Rajkumar, H.; Mahmood, N.; Kumar, M.; Varikuti, S.R.; Challa, H.R.; Myakala, S.P. Effect of probiotic (vsl#3) and omega-3 on lipid profile, insulin sensitivity, inflammatory markers, and gut colonization in overweight adults: A randomized, controlled trial. *Mediat. Inflamm.* **2014**, *2014*, 348959.
14. Ranganathan, N.; Friedman, E.A.; Tam, P.; Rao, V.; Ranganathan, P.; Dheer, R. Probiotic dietary supplementation in patients with stage 3 and 4 chronic kidney disease: A 6-month pilot scale trial in Canada. *Curr. Med. Res. Opin.* **2009**, *25*, 1919–1930.
15. Sharma, B.; Srivastava, S.; Singh, N.; Sachdev, V.; Kapur, S.; Saraya, A. Role of probiotics on gut permeability and endotoxemia in patients with acute pancreatitis: A double-blind randomized controlled trial. *J. Clin. Gastroenterol.* **2011**, *45*, 442–448.
16. Stiksrud, B.; Nowak, P.; Nwosu, F.C.; Kvale, D.; Thalme, A.; Sonnerborg, A.; Ueland, P.M.; Holm, K.; Birkeland, S.E.; Dahm, A.E.; et al. Reduced levels of *d*-dimer and changes in gut microbiota composition after probiotic intervention in hiv-infected individuals on stable art. *J. Acquir. Immune Defic. Syndr.* **2015**, *70*, 329–337.
17. Tan, M.; Zhu, J.C.; Du, J.; Zhang, L.M.; Yin, H.H. Effects of probiotics on serum levels of Th1/Th2 cytokine and clinical outcomes in severe traumatic brain-injured patients: A prospective randomized pilot study. *Crit. Care* **2011**, *15*, R290.
18. Valentini, L.; Pinto, A.; Bourdel-Marchasson, I.; Ostan, R.; Brigidi, P.; Turroni, S.; Hrelia, S.; Hrelia, P.; Bereswill, S.; Fischer, A.; et al. Impact of personalized diet and probiotic supplementation on inflammation, nutritional parameters and intestinal microbiota—The “ristomed project”: Randomized controlled trial in healthy older people. *Clin. Nutr.* **2015**, *34*, 593–602.
19. Villar-Garcia, J.; Hernandez, J.J.; Guerri-Fernandez, R.; Gonzalez, A.; Lerma, E.; Guelar, A.; Saenz, D.; Sorli, L.; Montero, M.; Horcajada, J.P.; et al. Effect of probiotics (saccharomyces boulardii) on microbial translocation and inflammation in HIV-treated patients: A double-blind, randomized, placebo-controlled trial. *J. Acquir. Immune Defic. Syndr.* **2015**, *68*, 256–263.
20. Zarrati, M.; Salehi, E.; Nourijelyani, K.; Mofid, V.; Zadeh, M.J.; Najafi, F.; Ghaflati, Z.; Bidad, K.; Chamari, M.; Karimi, M.; et al. Effects of probiotic yogurt on fat distribution and gene expression of proinflammatory factors in peripheral blood mononuclear cells in overweight and obese people with or without weight-loss diet. *J. Am. Coll. Nutr.* **2014**, *33*, 417–425.
